# Gastrointestinal helminths of opossums (Mammalia: Didelphidae) from Bolivia

**DOI:** 10.1017/S0031182024000490

**Published:** 2024-06

**Authors:** F. Agustín Jiménez, Mariel L. Campbell, Beth Byles, Raymond Philip Scheibel, Scott L. Gardner

**Affiliations:** 1 School of Biological Sciences, Zoology, Southern Illinois University, Carbondale, IL, USA; 2 Museum of Southwestern Biology, 1 University of New Mexico, Albuquerque, NM, USA; 3 The Harold W. Manter Laboratory of Parasitology, University of Nebraska, Lincoln, NE, USA

**Keywords:** biological inventories, Bolivia, mouse opossums, parasite collections, taxonomic impediment

## Abstract

A total of 32 taxa of helminths were recovered from 52 individuals corresponding to 17 species of didelphiomorph marsupials collected across Bolivia. From these, 20 taxa are registered for the first time in this landlocked South American country, including the cestode *Mathevotaenia bivittata*, and the nematodes *Moennigia* sp., *Travassostrongylus callis*, *Viannaia didelphis*, *V. hamata*, *V. metachirops*, *V. minispicula*, *V. philanderi*, *V*. *simplicispicula, V. skrjabini*, *V. viannai*, *Cruzia tentaculata*, *Monodelphoxyuris dollmeiri*, *Neohilgertia venusti*, *Pterygodermatites elegans*, *Pterygodermatites jeagerskioldi*, *Spirura guianensis, Gongylonemoides marsupialis, Turgida turgida* and *Trichuris reesali*. We report for the first time parasites for *Marmosops bishopi, Monodelphis emiliae*, *Monodeplhis glirina*, *Monodelphis sanctarosae*, *Monodelphis peruviana* and *Thylamys sponsorius* and document 38 new records of parasites infecting marsupials. Twenty-six taxa of helminths infect 2 or more species of didelphiomorph marsupials, with the exception of *Travassostrongylus callis*, *Viannaia didelphis*, *V. hamata*, *V. minispicula* and *V. hamate*, which infected individuals of a single species.

## Graphical abstract

**Figure:**
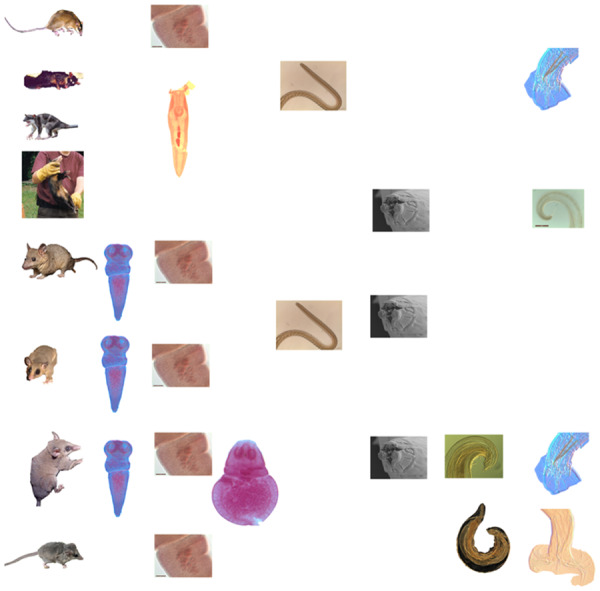

## Introduction

Didelphimorphia (opossums) is the most diverse order of marsupials present in the New World with over 125 extant species (Gardner, 2007; Teta *et al*., 2009; Voss and Jansa, 2009; Gutiérrez *et al*., 2010; Rossi *et al*., 2010; Jansa *et al*., 2014; Voss, 2022). This group includes arboreal, terrestrial, semiaquatic and scansorial representatives (Astúa, 2009; Flores, 2009; Voss and Jansa, 2009). Most of them occur in moderate densities in non-disturbed forests throughout the Neotropics (Lima *et al*., 2001; Gentile *et al*., 2004; Püttker *et al*., 2008; Pires *et al*., 2010). Several aspects of their ecology remain unknown, perhaps, because a large number of species is arboreal making their collection infrequent (Fontúrbel and Jiménez, 2009; Caceres *et al*., 2011; José *et al*., 2019). The territory of Bolivia sits at the juncture of high-altitude desserts, mountainous, temperate, tropical forest and lowland savannah biomes. This results in a large diversity of mammals that includes 24% of the extant species of opossums (Anderson, 1997; Gutiérrez *et al*., 2010; Rossi *et al*., 2010; Voss *et al*., 2012). This diversity includes representatives of all 4 subfamilies and major clades in the Didelphidae Gray and species that inhabit humid and dry forests (Voss and Jansa, 2009; Jansa *et al*., 2014; Voss, 2022).

From 1984 to 2000, the American Museum of Natural History (AMNH, New York City, USA), the Mammal Division of the Museum of Southwestern Biology (MSB:Mamm, University of New Mexico, Albuquerque New Mexico, USA), the Harold W. Manter Laboratory of Parasitology (HWML, University of Nebraska-Lincoln, Lincoln Nebraska, USA), and the Bolivian National Museum of Natural History in La Paz (Colección Nacional de Fauna Sección Mastozoología, CBF, La Paz, Bolivia) mounted joint US National Science Foundation funded collecting expeditions throughout Bolivia to survey and inventory sylvatic mammals and their parasites. A major part of the work on the mammals has been published by Anderson (1997) and many groups of parasites that were collected from these mammals are still being studied in earnest. From most of the mammals that were collected by these expeditionary research teams in Bolivia, data on habitat, habits and biological associates were also collected and archived in museums. All of the parasites are on deposit in the HWML within the Bolivian Mammal Parasite Collection (BMPC). The BMPC includes all specimens of helminths and parasitic arthropods that were recovered from the more than 16 000 mammals collected and preserved in museums during the course of our work.

Species of helminths that occur in opossums can be grouped in families that include opossum dwelling species, such as Rhopaliidae (Radev *et al*., 2005; Haverkost and Gardner, 2008) or in families that include species occurring in distantly related groups of mammals, i.e. Aspidoderidae, Onchocercidae, Viannaidae (Brant and Gardner, 2000; Jiménez *et al*., 2012; Scheibel *et al*., 2014). However, the establishment of their specificity or host range is difficult to assess without the existence of a database that relates the distribution of parasite species across several species of mammals. Herein, we present the recorded species of gastrointestinal helminths infecting opossums in Bolivia.

## Materials and methods

All mammals were collected using Sherman™ live traps baited with a mixture of oatmeal, vanilla, tuna and sardines, or with snap traps baited with peanut butter. Traps were placed in suitable habitat each evening and checked at first daylight the following morning. Details of each mammal collected were recorded in a field-collection catalog book and in the trap data book, copies of which are maintained in the HWML, the originals are in the Department of Mammalogy, AMNH. Mammal voucher specimens are deposited in the AMNH, MSB:Mamm and CBF.

In the field, each organ of the digestive system was examined separately. Platyhelminths found were placed in distilled water until they relaxed and were killed and fixed in either 70% EtOH or hot or cold 10% formalin. Nematodes were either placed directly in 70% ethanol or killed with glacial acetic acid then transferred to either 70% ethanol or 10% formalin solution. Some samples were preserved in 95% ethanol or in liquid nitrogen and then stored in −85°C freezers in the Manter Laboratory Parasite Genomic Research Facility.

Digenetic trematodes, cestodes and acanthocephalans were stained in Semichon's acetocarmine, dehydrated in a graded series of ethanol, cleared in xylene and mounted in Canada balsam or Damar Gum. Nematodes were cleared in lactophenol and mounted on temporary slides. Vouchers for this study were deposited in the HWML. Specimens used for comparison were borrowed from the HWML and 7 additional institutions including:

- CHIOC: *Coleção Helmintológica do Instituto Oswaldo Cruz*, Oswaldo Cruz Institute, Rio de Janeiro Brazil.
- CHLP: Collection of Helminths of the Division of Invertebrates of the Museum of Natural History of the National University of La Plata.
- CMNA: Canadian Museum of Nature Parasite Collection, Ottawa, Canada
- CNHE: Colección Nacional de Helmintos of the National Autonomous University of Mexico, Mexico City.
- CHIAUMSM: Colección de Helmintos e Invertebrados Afines, Museo de Historia Natural, Universidad Nacional Mayor de San Marcos, Lima.
- UCDNC: University of California Davis Nematode Collection, University of California, Davis, USA.
- USNPC: United States National Parasite Collection of the Smithsonian Institution. Washington D.C. U.S.A.

The list follows current systems of classification (Radev *et al*., 2005; Beveridge *et al*., 2014; Mariaux *et al*., 2017; Hodda, 2022). The helminthological record for most of the marsupials examined is available at http://opensiuc.lib.siu.edu/zool_data/23/. The relation of voucher specimens, numbers and collections is presented below.

## Results

A total of 32 taxa of helminths were recovered from 17 species of marsupials collected from 23 localities. The total includes 3 species of digenetic trematodes, 6 species of tapeworms, 22 species of nematodes, 2 of which remain unidentified and an acanthocephalan. The association of parasites and their didelphiomorph hosts is detailed in Tables 1–3. We present this association using the tribe of the mammals.

**Table 1.** Helminthological records in Bolivia for species of marsupials included in the tribe Thylamini

	*Marmosops bishopi* (*n* = 1)	*Marmosops ocellatus* (*n* = 1)	*Marmosops noctivagus* (*n* = 4)	*Thylamys pallidior* (*n* = 2)	*Thylamys venustus* (*n* = 5)	*Thylamys sponsorius* (*n* = 2)	*Thylamys* sp. (*n* = 3)
*Linstowia schmidti*					USNPC82216		
*Atriotaenia* sp.			HWML49845 (25%)		HWML70016–18 (20%)	HWML 118760, 118784 (100%)	
*Mathevotaenia bivittata*							HWML118719 (33%)
*Mathevotaenia sanmartini*				HWML70016–19 (100%)			
*Mathevotaenia* sp.	HWML 118786				HWML 118787 (20%)		HWML 118788 (33%)
*Pritchardia boliviensis*		HWML118789	HWML118790 and 118791 (50%)				
*Neohilgertia venusti*					HWML118792 (60%)		HWML61553 (66%)
*Pterygodermatites elegans*	HWML118745						
*Gongylonemoides marsupialis*					HWML118750 (20%)		
*Moennigia* sp.			HWML118766 and 118767 (25%)				
*Viannaia metachirops*			HWML 118724				
*Viannaia minispicula*			HWML 118726				
*Viannaia philanderi*					HWML1187728		
*Viannaia simplicispicula*					HWML118761–63	HWML118760	HWML118759 (33%)
*Viannaia viannai*			HWML 118734–36				HWML118794 (66%)
*Turgida turgida*	HWML118795		HWML1187757 (75%)				
*Trichuris reesali*			HWML118757 (25%)				

Collection number for a representative and prevalence are provided.

**Table 2.** Helminthological records in Bolivia for species of marsupials included in the tribe Marmosini

	*Monodelphis domestica* (*n* = 9)	*Monodelphis sanctarosae* (*n* = 1)	*Monodelphis glirine* (*n* = 1)	*Monodelphis peruviana* (*n* = 1)	*Monodelphis adusta* (*n* = 1)	*Marmosa* sp. (*n* = 4)
*Rhopalias*	HWML118796 and 118797 (22%)					
*Paralinstowia schmidti*	HWML38846 (11%)					
*Mathevotaenia bivittata*	HWML118798 (22%)					HWML118799 (50%)
*Pritchardia boliviensis*	HWML118800 (11%)					HWML118801 (50%)
*Didelphoxyuris* sp.						HWML118755 (25%)
*Monodelphoxyuris dollmeiri*	HWML60229, 60130 (22%)					
*Aspidodera raillieti*	HWML60236 (11%)	HWML118802				HWML118803 (25%)
*Spirura guianensis*	HWML118756 (33%)					
*Pterygodermatites elegans*						HWML 118744 (50%)
*Pterygodermatites jeagerskioldi*	HWML 118746 and 118747 (22%)					HWML 118744 (25%)
*Moennigia* sp.	HWML 118764 (33%)		HWML 118765			
*Viannaia viannai*	HWML118805 (33%)			HWML 118737	HWML118806	HWML 118732 and 118733
*Trichuris reesali*						HWML118807 (25%)

*Monodelphis kunsi* was infection free. Collection number for a representative and prevalence are provided.

**Table 3.** Helminthological records in Bolivia for species of marsupials included in the tribe Didelphini

	*Didelphis albiventris* (*n* = 3)	*Didelphis marsupialis* (*n* = 3)	*Chironectes minimus* (*n* = 2)	*Philander opossum* (*n* = 7)
*Rhopalias caballeroi*			HWML70021 (50%)	
*Rhopalias coronatus*				HWML70000, HWML70002; HWML70009 (42%)
*Rhopalias macracanthus*				HWML70001; HWML70003; HWML70010; HWML70028 (57%)
*Mathevotaenia bivittata*				HWML118808 (28.6%)
*Pritchardia boliviensis*				HWML118809 (14.3%)
*Didelphoxyuris thylamisis*				
*Aspidodera raillieti*	HWML118738 (33%)	HWML61882 and 118810 (100%)		HWML118741 (28.6%)
*Turgida turgida*	HWML118751 (33%)	HWML118811 (75%)		
*Cruzia tentaculata*	HWML118741 and 118742 (33%)			HWML118743 (14.3%)
*Spirura guianensis*			HWML 118748 (50%)	
*Travassostrongylus callis*			HWML 118721 (50%)	
*Viannaia hamata*		HWML 118723		
*Viannaia simplicispicula*		HWML 118758		
*Viannaia skrjabini*		HWML 118729		
*Viannaia viannai*		HWML 118730		HWML118812 (43%)
*Oligacanthorhynchus microcephala*		HWML60388		HWML60600

Collection number for a representative and prevalence are provided.

### Phylum Platyhelminthes Gegenbaur, 1859

#### Class Trematoda Rudolphi, 1808 Order Diplostomida Olson, Cribb, Tkach, Bray and Littlewood, 2003 Superfamily Echinostomoidea Looss, 1902 Family Rhopaliidae Looss, 1899 *Rhopalias* Stiles and Hassall, 1898 1. *Rhopalias caballeroi* Kifune and Uyema, 1982

*Site of infection*: Small intestine

*Type host and locality*: *Didelphis marsupialis* L., Huanuco, Peru

*Other reported hosts*: *Chironectes minimus* (Zimmermann), *Didelphis* sp., *Philander opossum* (L.), *Lutreolina crassicaudata* (Desmarest)

*Locality records*: Argentina: Buenos Aires, Berisso. Colombia: undetermined. Mexico: Veracruz: Los Tuxtlas. Panama: Panama Canal. Peru: Cusco: Pilcopata; San Martín: Bella Vista; Llamas. Venezuela: Aragua (Tantaleán and Chavez, 2004; Haverkost and Gardner, 2008; Chero *et al*., 2017)

*Records in Bolivia*: *Chironectes minimus*: La Paz: La Reserva, 15°44′S, 67°31′W, 850 m, 22 July 1992, HWML70021 (274 specimens) from MSB:MAMM:68330. *Didelphis marsupialis*: Santa Cruz: San Rafael de Amboró, 17°21′S, 63°43′W, 400 m, 24 July 1985, HWML70025 (3 specimens) from MSB:MAMM:55833. *Philander opossum*: Santa Cruz: San Miguel del Rincón, 17°23′S, 63°32′W, 300 m; 13 August 1984, HWML70018 (3 specimens) from MSB:MAMM:55074; Estancia Cachuela Esperanza, 16°46′59.99″S, 63°13′59.99″W, 300 m, 22 August 1984, HWML70021 (18 specimens) from MSB:MAMM:210569.

*Additional specimens examined*: HWML70014 (1 specimen) from *Lutreolina crassicaudata*, Berisso, Buenos Aires, Argentina; CNHE 4081 (1 specimen) from *Didelphis* sp., Catemaco, Veracruz, Mexico; CNHE965 (1 specimen) from *Didelphis marsupialis*, Aragua, Venezuela.

#### 2. *Rhopalias coronatus* (Rudolphi, 1819) Stiles and Hassall, 1898

*Synonyms*: *Rhopalias dobbini* Prod'Hon, 1968

*Site of infection*: Small intestine

*Type host and locality*: *Didelphis marsupialis*, Brazil.

*Other reported hosts*: *Didelphis albiventris* Lund, *Didelphis pernigra* (J.A. Allen), *Lutreolina crassicaudata*, *Metachirus myosurus* (Temminck) and *Philander opossum*.

*Locality records*: Argentina: Buenos Aires: Berisso. Brazil: Bahía: Igrapiúna; Minas Gerais: Belo Horizonte. Costa Rica: Cariai. Mexico: Chiapas: Motozintla; Oaxaca: Cuicatlán; Quintana Roo: La Ceiba; Veracruz: Alvarado and Los Tuxtlas. Panama: Canal Zone. Paraguay: undetermined. Peru: Ancash: Marca; Cajamarca: undetermined; Huánuco: undetermined; Pasco: Villa Rica; San Martin: Llamas. Venezuela: El Tacal (Siebert, 1970; Silva and Costa, 1999; Haverkost and Gardner, 2008; Chero *et al*., 2017; Polo-Gonzales *et al*., 2019; Cirino *et al*., 2020).

*Records in Bolivia*: *Philander opossum*: Santa Cruz: Estancia Cachuela Esperanza, 16°47″, 63°14′, 300 m, 22 August 1984, HWML70000 (108 specimens) from MSB:MAMM:210569; 15 km S of Santa Cruz, 17°53′S, 63°07′W, 2 August 1987, HWML70002 (39 specimens), from AMNH263965; 3 km SE of Montero, 1 km N of Villa Copacabana, 17°23′S, 63°14′W, 250 m, 26 June 1991, HWML70009 (21 specimens), from AMNH263963.

*Additional specimens examined*: HWML34950 (1 specimen) from *Didelphis albiventris*, Paraguay. HWML70013 (1 specimen) from *Lutreolina crassicaudata*, Berisso, Buenos Aires, Argentina.

#### 3. *Rhopalias macracanthus* Chandler, 1932

*Synonyms*: *Rhopalias louisiana* Hearin, 1937

*Site of infection*: Small intestine

*Type host and locality*: *Didelphis virginiana* Kerr, Houston, Texas

*Other reported hosts*: *Didelphis marsupialis*.

*Locality Records*: Costa Rica: Cariai. Mexico: Colima: Comala, La Esperanza. Chiapas: Jaltengo, Motozintla, and Pueblo Nuevo; Oaxaca: Temazcal; Quintana Roo: Rancho La Ceiba; Veracruz: Alvarado, and Los Tuxtlas. United States: Florida: Tallahasee; Illinois: Jackson Co.; Maryland: Beltsville; Texas: Houston (Siebert, 1970; Alden, 1995; Haverkost and Gardner, 2008).

*Records in Bolivia*: *Philander opossum*: Santa Cruz: Santa Cruz, 16°28′12″S, 63°08′24″W, HWML70001 (1 specimen); 15 km S of Santa Cruz, 17°53′S, 63°07′W, 2 August 1987, HWML70003 (7 specimens), from AMNH263965 and 263966; 3 km SE of Montero, 1 km N of Villa Copacabana, 17°23′S, 63°14′W, 250 m, 26 June 1991, HWML70010, (3 specimens) from AMNH263963; 10 km N of San Ramón, 16°36′S, 62°42′W, 250 m, 7 August 1985, HWML70028 (1 specimen), from host MSB:MAMM:55857.

*Additional specimens examined*: USNPC8548 (1 specimen) from *Didelphis marsupialis*, Houston.

Remarks: *Rhopalias macracanthus* and *R*. *coronatus* cause co-infections in the Gray four-eyed opossum, *Philander opossum*. Haverkost and Gardner (2008) reviewed species in the family across the continent, making observations and identifying reliable characters based on morphometric analyses.

#### Class Cestoda Rudolphi, 1808 Order Cyclophyllidea van Beneden in Braun, 1900 Family Anoplocephalidae Blanchard, 1891 Subfamily Linstowiinae Fuhrmann, 1907 *Mathevotaenia* Akhumyan, 1946 4. *Mathevotaenia bivittata* (Janicki, 1904) Yamaguti, 1959

*Synonyms: Oochoristica bivittata* Janicki, 1904; *Linstowia* (*Opossumia*) *bivittata* (Janicki, 1904) Spasskii, 1951; *Opossumia bivittata* Spasskii, 1981.

*Site of infection*: Small intestine

*Type host and locality*: *Marmosa* sp., Brazil.

*Other reported hosts*: *Caluromys derbianus* (Waterhouse), *Didelphis albiventris*, *Didelphis marsupialis*, *Marmosa paraguayana* Tate (as *Micoreus cinereus*), *Marmosa murina* (L.), *Marmosa demerarae* (Thomas), *Metachirus nudicaudatus* (É. Geoffreoy), *Monodelphis domestica* (Wagner), *Philander opossum*, and *Thylamys* sp.

*Locality records*: Argentina: Salta: Orán. Brazil: Pará: Belém, Bassuquara and Bacia de Agua Preta; Mato Grosso do Sul: Bodoquena; Espirito Santo: Santa Teresa; Rio de Janeiro: Angra dos Reis. Panama: Canal Zone. Trinidad and Tobago: Rio Claro, Sangre Grande. French Guiana: Cayenne, Nouragues, Saut Pararé and Saül, Pic Matecho (Foster, 1939; dos Santos, 1968; Campbell *et al*., 2003; Byles *et al*., 2013).

*Records in Bolivia*: *Thylamys* sp.: Tarija: 3 km S of Cuyambuyo, 22°16′S, 64°33′W, 900 m, 3 and 4 August 1991, HWML118719 (207 specimens) from MSB:MAMM:240043.

*Additional specimens examined*: HWML 17712 from *Marmosa cinerea* (Temminck), Argentina, Salta, Orán. HWML 49769 from *Marmosa murina* French Guiana, Cayenne, Montagne du Tigre.

#### 5. *Mathevotaenia sanmartini* Jiménez, Braun, Campbell and Gardner, 2008

*Site of infection*: Small intestine

*Type host and locality*: *Thylamys pallidior* (Thomas), OMNH 34911, Argentina: Jujuy: Susques, 8.2 km south of Sey (by road), 24°00′48.8″S, 66°30′52.8″W, 4167 ± 10 m (31 March and 1 April 2006).

*Locality and host records*: No additional records available.

*Records in Bolivia*: *Thylamys pallidior*: Cochabamba: Curubamba, 7.5 km southeast of Rodeo (by road), 17°40′31″S, 65°36′04″W, 4000 m, 24 and 26 July 1993, HWML70016–19 (1 and 3 specimens) from MSB:MAMM:87100 and MSB:MAMM:87102.

*Additional specimens examined*: CHLP5727 (1 specimen) holotype from *Thylamys pallidior*, Argentina.

*Remarks*: Several of these specimens were reported in the original description of the species (Jiménez *et al*., 2008).

#### 6. *Mathevotaenia* sp.

*Site of infection*: Small intestine

*Type host and locality*: Not yet named.

*Records in Bolivia*: *Marmosops bishopi* (Pine): La Paz: La Reserva, 15°44′S, 67°31′W, 850 m, 27 July 1992, HWML118786 (1 specimen) from MSB:MAMM:235887. *Marmosops noctivagus* (Tschudi): Cochabamba: 9.5 km by road NE of Tablas Monte, Río Jatún Mayu; 17°2′S, 65°59′W, HWML49845 from MSB:MAMM:70278. *Thylamys pusillus* (Desmarest): Santa Cruz: 53 km E Boyuibe, 20°27′S, 62°50′W, 600 m, 6 July 1991, HWML 118788 (6 specimens) from MSB:MAMM:87105. *Thylamys venustus*: Tarija: Tapecua, 21°26′S, 63°55′W, 1500 m, 12 July 1991, HWML 118787 (2 specimens) from AMNH275439.

#### 7. *Paralinstowia schmidti* (Gardner and Campbell, 1992) Beveridge and Spratt, 2003

*Site of infection*: Small intestine

*Type host and locality*: *Thylamys elegans venusta* (Thomas), Bolivia: Chuquisaca, El Porvenir 20°45′S, 63°13′W, 675 m, 6 July, 1985, symbiotype: AMNH261257.

*Other reported hosts*: *Monodelphis domestica* (Wagner)

*Locality records*: None available.

*Records in Bolivia*: Chuqisaca: El Porvenir, 20°27′W, 63°07′48″S, 675 m, 15 July 1985, UCDNC2831 (32 specimens) from host MSB:MAMM:211200.

*Remarks*: Both *Monodelphis domestica* and *Thylamys elegans venusta* -junior synonym of *Thylamys venustus* (Thomas)- were the only marsupials collected in El Porvenir. The species was not found in the other three localities were specimens of *Monodelphis domestica* were collected.

#### 8. *Pritchardia boliviensis* Gardner, Jiménez and Campbell, 2013

*Site of infection: Small intestine*

*Type host and locality: Marmosops noctivagus*: Cochabamba: 9.5 km by road NE of Tablas Monte, Río Jatun Mayu 17°02′29″S, 65°59′05″W, 1500 m, 14 July 1993, symbiotype MSB:MAMM:70278.

*Other reported hosts*: *Marmosa paraguayana*, *Metachirus nudicaudatus*, *Gracilinanus* sp., *Marmosops ocellatus* (Tate)

*Locality records*: Brazil: Paraná: between Corbélia and Cascavel. Paraguay: Alto Paraná: Estación Biológica Limoy (Gardner *et al*., 2013; Benatti *et al*., 2023).

*Records in Bolivia*: *Marmosa* sp.: Santa Cruz: 53 km E of Boyuibe, 20°27′S, 62°50′W, 600 m, 8 July 1991, HWML118801 (139 specimens) from MSB:MAMM:239772. *Marmosops noctivagus*: Cochabamba: 9.5 km by road NE Tablas Monte, 17°02′S, 65°59″W, 14 and 16 July 1993, HWML118790 and HWML118791 (98 and 65 specimens) from MSB:MAMM:70278 and MSB:MAMM: 30279; La Paz: Chijchijpa, 16°09′S, 67°45′W, 1114 m, 8 July 1992, HWML61763 (19 specimens) from host MSB:MAMM:235553. *Marmosops ocellatus*: Santa Cruz: 3.5 km W, Estación El Pailón, 17°39′S, 62°45′W; 300 m, 21 September 1984, HWML118789 from MSB:MAMM:55070. *Metachirus nudicaudatus*: La Paz: La Reserva, 15°44′S, 67°31′W; 840 m. 24 July 1992, CNHE6422, CHIOC37318, USNPC103071, from CBF2310. *Monodelphis domestica*: Santa Cruz: 1 km S and 3 km W of Estancia Isibolos, 19°31′S, 63°36′W, 930 m, 5 July 1991, HWML118800 (52 specimens) from MSB:MAMM:239734. *Philander opossum*: Santa Cruz: 3 km SE Montero, 1 km N Villa Copacabana, 17°23′S, 63°14′W, 250 m, 26 June 1991, HWML118809 (1 specimen) from MSB:MAMM:239685.

*Remarks*: The holotype for this species was examined and used as a comparative reference.

#### *9. Atriotaenia* sp.

*Site of infection*: Small intestine

*Records in Bolivia*: *Marmosops noctivagus*: La Paz: La Reserva, 15°44′S, 67°31′W, 850 m, 24 July 1992, HWML118724 (4 specimens) from MSB:MAMM:235815. *Thylamys venustus*: Tarija: 3 km SE Cuyambuyo, 22°16′S, 64°33′W, 900 m, 4 August 1991, HWML118720 (2 specimens) from MSB:MAMM:140296. *Thylamys sponsorius* (Thomas): Tarija: 3 km SE Cuyambuyo, 22°16′S, 64°33′W, 900 m, 4 August 1991, HWML118760 (1 specimen) from MSB:MAMM:67014, HWML118784 (365 specimens from MSB:MAMM:67015).

*Remarks*: Most of the specimens were contracted, making it difficult to identify to species level.

### Phylum Nematoda Cobb, 1932

#### Class Chromadoria Pearse, 1936 Order Rhabditida Chitwood, 1933 Superfamily Ancylostomatoidea Looss, 1905 Family Ancylostomatidae Looss, 1905 Subfamily Bunostominae Railliet and Henry, 1909 *Monodontus* Molin, 1860 *10. Monodontus* sp.

*Site of infection*: Small intestine

*Records in Bolivia*: *Thylamys venustus*: Tarija: 3 km SE Cuyambuyo, 22°16′S, 64°33′W, 900 m, 4 August 1991, HWML118720 (1 specimen) from MSB:MAMM:140296.

*Remarks*: This is a single mature female. Species of the genus are known to typically infect rodents.

#### Superfamily Molineoidea Skrjabin and Shulz, 1937 Family Molineidae Skrjabin and Shulz, 1937 Subfamily Anoplostrongylinae Chandler, 1938 *Moennigia* Travassos, 1935 11. *Moennigia* sp.

*Site of infection*: Small intestine

*Records in Bolivia*: *Monodelphis domestica*: Chuquisaca: Río Limón, 19°33′S, 64°08′W, 1300 m, 3 August 1990 HWML118764 (1 specimen) from MSB:MAMM:63278. *Monodelphis glirina*: Pando: Santa Rosa, 12°07′48″S, 68°14′24″W, 800 m, 1 August 1986, HWML118765 (11 specimens) AMNH M 262399. *Marmosops noctivagus*: Cochabamba: 9.5 km by road NE Tablas Monte, 17°02′S, 65°59″W, 14 and 15 July 1993, HWML118766 and HWML118767 (1 specimen each) from MSB:MAMM:70278 and MSB:MAMM:238453.

*Remarks*: These individuals belong to a single species which may be new to science. The senior author is attempting to work in the precise identification and description.

#### Superfamily Heligmosomoidea Cram, 1927 Family Viannaiidae Neveu-Lemaire, 1944 Subfamily Viannaiinae Neveu-Lemaire, 1944 *Travassostrongylus* Orloff, 1933 12. *Travassostrongylus callis* (Travassos, 1914) Orloff, 1933

*Synonyms: Trichostrongylus callis* Travassos 1914*; Ostertagia callis* (Travassos, 1914) Travassos 1918

*Site of infection*: Small intestine

*Type host and locality*: *Didelphis aurita* (Wied-Neuwied), Brazil: Rio de Janeiro: Manguinhos CHIOC 724.

*Other reported hosts*: *Didelphis marsupialis*, *Philander opossum*.

*Locality records*: Brazil: Espirito Santo: Sooretama; Rio de Janeiro: Petrópolis. French Guiana: undetermined. Panama: Panama City (Diaw, 1976*a*, 1976*b*; Scheibel *et al*., 2014).

*Records in Bolivia*: *Chironectes minimus*: La Paz: La Reserva, 15°44′S, 67°31′W, 850 m, 22 July 1992, HWML118721 (6 specimens) from MSB:MAMM:68330.

*Additional specimens examined*: From *Didelphis aurita*, Brazil: Rio de Janeiro: CHIOC 8426, 8584, 8589, 9608 Manguinhos; CHIOC 9118 Petrópolis; CHIOC 29504, 29505 Espirito Santo: Sooretama.

#### *Viannaia* Travassos, 1914 13. *Viannaia didelphis* (Travassos, 1914) Durette-Desset, 1968

*Synonyms: Nematodirus* (*Mecistocirrus*) *didelphis* Travassos, 1914

*Site of infection*: Small intestine

*Type host and locality*: *Didelphis aurita*, Brazil, Rio de Janeiro, Manguinhos CHIOC 942.

*Other reported hosts*: *Didelphis marsupialis* and *Didelphis virginiana*.

*Locality records*: Costa Rica: Guanacaste, Colonia Bolaños. Mexico: Colima: La Esperanza, Madrid. Panama: Panama City. United States: Georgia: Enigma, Bulloch Co.; Louisiana: Jeanerette; Illinois: Urbana, Jackson Co.; North Carolina: undetermined; Tennessee: Reelfoot Lake. Trinidad and Tobago: undetermined; Venezuela: Maracaibo (Guerrero, 1985; Alden, 1995; Monet-Mendoza *et al*., 2005; Scheibel *et al*., 2014).

*Records in Bolivia*: *Marmosa* sp.: La Paz: Chijchijpa, 16°09′S, 67°45′W, 1114 m, 8 July 1992, HWML118722, HWML61763 from host MSB:MAMM:235553.

*Remarks*: Species in *Viannaia* have been reported in several species of marsupials across the Americas (Dikmans, 1931; Cañeda-Guzmán, 1997; Ellis *et al*., 1999; Silva and Costa, 1999; Gomes *et al*., 2003; Antunes, 2005; Byles *et al*., 2013). A few studies suggest some infections are caused by multiple species (Diaw, 1976*a*; Guerrero, 1985; Scheibel *et al*., 2014), thus, individual identification of these nematodes is recommended.

#### 14. *Viannaia hamata* Travassos, 1914

*Site of infection*: Small intestine

*Type host and locality*: *Didelphis aurita*, Brazil, Rio de Janeiro, Manguinhos CHIOC 942

*Other reported hosts*: *Didelphis albiventris*, *Didelphis marsupialis, Didelphis virginiana*, *Philander opossum*, *Marmosa cinerea*, *Marmosa murina*.

*Locality records*: Brazil: Minas Geráis, Belo Horizonte; Pará: Belém; Río de Janeiro: Glicêrio, Petrópolis; Paraná: between Corbélia and Cascavel; Río Grande do Sul: Pelotas; Peru: Pasco: Villa Rica; San Martín: Bella Vista, Lamas. Trinidad and Tobago, undetermined; United States: Georgia, Macintosh Co., Bulloch Co.; North Carolina. Venezuela: Miranda, Guatopo (Wolfgang, 1951; Guerrero, 1985; Ellis *et al*., 1999; Silva and Costa, 1999; Gomes *et al*., 2003; Chero *et al*., 2017; Polo-Gonzales *et al*., 2019; Benatti *et al*., 2023).

*Records in Bolivia*: *Didelphis marsupialis*: La Paz: La Reserva, 15°44′S, 67°31′W, 850 m, 22 July 1992, HWML118723 (2 specimens) from MSB:MAMM:235674.

*Additional specimens examined*: CHIOC 29289 and 29290 from *Didelphis* sp. Brazil, Rio de Janeiro, Usina da Tijuca.

#### 15. *Viannaia metachirops* Durette-Desset, 1974

*Site of infection*: Small intestine

*Type host and locality*: *Philander opossum*, French Guiana

*Other reported hosts*: None available

*Locality records*: None available

*Records in Bolivia*: *Marmosops noctivagus*: La Paz: La Reserva, 15°44′S, 67°31′W, 850 m, 24 July 1992, HWML118724 (5 specimens) from MSB:MAMM:235815. *Marmosa* sp., La Paz: Chijchijpa, 16°09′S, 67°45′W, 1114 m, 8 July 1992, HWM118725 (3 specimens) from host MSB:MAMM:235553.

*Additional specimens examined*: None, identification made based on diagnostic traits.

#### 16. *Viannaia minispicula* Guerrero, 1985

*Site of infection*: Small intestine

*Type host and locality*: *Marmosa murina*, Venezuela: Amazonas, Caño Yaguá

*Other reported hosts*: *Marmosa demerarae*, *Philander opossum*

*Locality records*: French Guiana: Guyanne, Cacao.

*Records in Bolivia*: *Marmosops noctivagus*: Cochabamba: 9.5 km by road NE Tablas Monte, 17°02′S, 65°59″W, 15 July 1993, HWML118726 (1 specimen) from MSB:MAMM:238453.

*Additional specimens examined*: None, identification made based on diagnostic traits.

#### 17. *Viannaia philanderi* (Wolfgang, 1951) Durette-Desset, 1968

*Site of infection*: Small intestine

*Type host and locality*: *Caluromys philander*, Trinidad.

*Other reported hosts*: None available

*Locality records*: None available

*Records in Bolivia*: *Marmosa* sp.: Santa Cruz: Estancia Cachuela Esperanza, 16°46′59.99″S, 63°13′59.99″W, 300 m, 24 August 1984, HWML118727 (3 specimens) from MSB:MAMM:211050. *Thylamys venustus*: Tarija: 3 km SE Cuyambuyo, 22°16′S, 64°33′W, 900 m, 4 August 1991, HWML118728 (13 specimens) from MSB:MAMM:140297.

*Additional specimens examined*: None, identification made based on diagnostic traits.

#### 18. *Viannaia simplicispicula* (Navone, Suriano and Pujol, 1991) Jiménez *et al*., 2024

*Synonyms*: *Hoineffia simplicispicula* Navone, Suriano and Pujol, 1991

*Site of infection*: Small intestine

*Type host and locality*: *Thylamys venustus cinderellus* (Thomas 1902): Argentina: Tucumán: Quebrada Los Sosa, Museo Argentino Bernardino Rivadavia No. 360.

*Other reported hosts*: *Tlacuatzin canescens* (J.A. Allen).

*Locality records*: Argentina: Jujuy: Dr Manuel Belgrano, Las Capillas and El Palmar; Salta: Mosconi. Mexico: Oaxaca: Santa Catarina Juquila (Jiménez *et al*., 2008; Guzmán-Cornejo *et al*., 2012).

*Records in Bolivia*: *Didelphis marsupialis*: La Paz: La Reserva, 15°44′S, 67°31′W, 850 m, 22 July 1992, HWML118758 (1 specimen) from MSB:MAMM:235674. *Thylamys* sp.: Tarija: 3 km S of Cuyambuyo, 22°16′S, 64°33′W, 900 m, 4 August 1991, HWML118759 (18 specimens) from MSB:MAMM:240043. *Thylamys sponsorius*: Tarija: 3 km SE Cuyambuyo, 22°16′S, 64°33′W, 900 m, 4 August 1991, HWML118760 (1 specimen) from MSB:MAMM:67015. *Thylamys venustus*: Tarija: 3 km SE Cuyambuyo, 22°16′S, 64°33′W, 900 m, 4 August 1991, HWML118761 (18 specimens) from MSB:MAMM:140296, HWML118762 (10 specimens) from MSB:MAMM:140297; Tapecua, 21°26′S, 63°55′W, 1500 m, 12 July 1991, HWML118763 (4 specimens) from AMNH275439.

*Remarks:* Guerrero (1985) transferred *Hoineffia cayennensis* Diaw, 1976 to *Viannaia*. This recommendation was based on the observation that the transversally elongated bursa – diagnostic for *Hoineffia* Diaw, 1976 – also occurs in other species featuring a gubernaculum such as *Viannaia venezuelensis* Guerrero, 1985 and *Viannaia barusi* Guerrero, 1985. Furthermore, other species in the genus feature the combination of cordiform bursa and lack of gubernaculum, such as *Viannaia viannai*. The phenotypic plasticity of the bursa is shown in a subset of species of *Viannaia* collected across Mexico (Ramírez-Cañas *et al*., 2021). *Hoinneffia simplicispicula* Navone, Suriano and Pujol, 1991 was proposed as the second species in the genus; further, the species was recorded in in Mexico and in Argentina (Jiménez *et al*., 2008; Guzmán-Cornejo *et al*., 2012). Apparently, Navone *et al*. (1991) were not familiar with the change proposed by Guerrero (1985). We herein consider that the differences in the shape of the caudal bursa, the relative length of the dorsal lobe, dorsal ray and ray 8 are consistent with the intraspecific variability documented by Guerrero (1985). Further this variability is observed in specimens from Argentina, Bolivia and French Guiana. Rather than proposing an amended diagnosis, we refer readers to the diagnosis proposed by Dikmans (1945), who only missed the presence of three ventral ridges proposed by Durette-Desset (1971) in his definition of the genus.

*Additional specimens examined*: HWML63395 from *Thylamys venustus*, 24.8 km N of Santa Clara (by road), Jujuy, Argentina.

#### 19. *Viannaia skrjabini* Lent and Freitas, 1937

*Site of infection*: Small intestine

*Type host and locality*: *Philander opossum*, Brazil: Río de Janeiro: Petrópolis. CHIOC 7721

*Other reported hosts*: *Didelphis albiventris*, *Didelphis marsupialis* and *Marmosa robinsoni* Bangs.

*Locality records*: Brazil: Pernambuco, Exu. Venezuela: Amazonas, Caño Yaguá; Miranda: Río Negro; Distrito Federal: Naiguatá (Guerrero, 1985).

*Records in Bolivia*: *Didelphis marsupialis*: La Paz: La Reserva, 15°44′S, 67°31′W, 850 m, 22 July 1992, HWML118729 (from HWML61838) (3 specimens) from MSB:MAMM:235674.

*Additional specimens examined*: Holotype CHIOC7721, from *Philander opossum*, Brazil: Río de Janeiro, Petrópolis

#### 20. *Viannaia viannai* Travassos, 1914

*Site of infection*: Small intestine

*Type host and locality*: *Didelphis aurita*, Brazil, Rio de Janeiro, Manguinhos CHIOC 922.

*Other reported hosts*: *Didelphis marsupialis*, *Didelphis virginiana*, *Philander opossum*.

*Locality records*: Brazil: Pernambuco: Exu; Rio de Janeiro: Morro São João, Casimiro de Abreu. Costa Rica: Guanacaste, Colonia Baños. French Guiana: Camp du Tigre. Mexico: Guerrero: Taxco El Viejo. Panama: Panama City. Peru: San Martín: Bella Vista. United States: Illinois, Carbondale; Maryland, Beltsville. Venezuela: Miranda: Caño Yagua, Río Negro and San Antonio (Guerrero, 1985; Monet-Mendoza *et al*., 2005; Scheibel *et al*., 2014; Chero *et al*., 2017).

*Records in Bolivia*: *Didelphis marsupialis*: La Paz: Chijchijpa, 16°09′S, 67°45′W, 1114 m, 6 July 1992, HWML118730 (1 specimen) from MSB:MAMM:235570; La Reserva, 15°44′S, 67°31′W, 850 m, 22 July 1992, HWML118731 (1 specimen) from MSB:MAMM:235674. *Marmosa* sp.: La Paz: Chijchijpa, 16°09′S, 67°45′W, 1114 m, 8 July 1992, HWML118732 (3 specimens) from host MSB:MAMM:235553; Santa Cruz: Estancia Cachuela Esperanza, 16°46′59.99″S, 63°13′59.99″W, 300 m, 24 August 1984, HWML118733 (7 specimens) from MSB:MAMM:211050. *Marmosops noctivagus*: Cochabamba: 9.5 km by road NE Tablas Monte, 17°02′S, 65°59″W, 14, 15 and 16 July 1993, HWML62620, HWML118734–36 (11, 13 and 10 specimens) from MSB:MAMM:70278, MSB:MAMM:238453 and MSB:MAMM:30279; La Paz: La Reserva, 15°44′S, 67°31′W, 850 m, 24 July 1992, HWML61852 (10 specimens) from MSB:MAMM:235815. *Monodelphis peruviana*: La Paz: La Reserva, 15°44′S, 67°31′W, 850 m, 25 July 1992, HWML118737 (3 specimens) from MSB:MAMM:68336. *Thylamys* sp.: Tarija: Tapecua, 21°26′S, 63°55′W, 1500 m, 1 June 1991, HWML118794 (5 specimens) from MSB:MAMM:238757.

*Additional specimens examined*: From *Didelphis marsupialis*, HWML67179–81, Panama City, Panama and Colonia Baños, Costa Rica. From *Didelphis virginiana* HWML61798 from Carbondale Illinois, U.S.A.

#### Order Spirurida Railliet, 1915 Suborder Ascaridina Inglis, 1983 Superfamily Heterakoidea Railliet and Henry, 1912 Family Aspidoderidae Skrjabin and Shikhobalova, 1947 *Aspidodera* Railliet and Henry, 1912 21. *Aspidodera raillieti* Travassos, 1913

*Synonyms*: *Aspidodera harwoodi* Chandler, 1932, *Aspidodera vicentei* Pinto, Kohn, Fernandes and Mello, 1981, *Aspidodera diaz-ungriai* Masí-Pallarés and Benítez-Uscher, 1971

*Site of infection*: Caecum and large intestine

*Type host and locality*: *Didelphis aurita*, Manguinhos, Brazil

*Other reported hosts*: *Didelphis marsupialis*, *Didelphis virginiana*, *Didelphis pernigra*, *Gracilinanus agilis* (Burmeister), *Marmosa demerarae*, *Marmosa murina*, *Marmosops ocellatus*, *Metachirus nudicaudatus*, *Metachirus myosurus*, *Philander opossum*, Sigmodontinae: *Nectomys squamipes* (Brants), *Euoryzomys nitidus* (Thomas).

*Locality records*: Brazil: Bahía: Igrapiúna; São Paulo, Piauí, Formosa. French Guiana: Montagne du Tigre, Nouragues, Saül, Petit Saut, Route de Kaw. Guatemala: Santa Rosa. Mexico: Motozintla; Panama: Panama Canal. Paraguay: Puerto Ibapobó. Suriname. Peru: La Libertad, Bosque del Cachil; San Martín: Bella Vista; Llamas. United States: Texas, Houston; Illinois, Jackson Co., Union Co., (Santos *et al*., 1990; Alden, 1995; Jiménez-Ruiz *et al*., 2008; Chero *et al*., 2017; Polo-Gonzales *et al*., 2019; Varella *et al*., 2022)

*Records in Bolivia*: *Didelphis albiventris*: Tarija: Tapecua, 21°26′S, 63°55′W, 1500 m, 14 July 1991, 1500 m, HWML118738 (36 specimens) from CBF2379. *Didelphis marsupialis*: La Paz: La Reserva, 15°44′S, 67°31′W, 850 m, 22 and 25 July 1992, HWML118810 (7 specimens) from MSB:MAMM:235674, HWML61882 (2 specimens) from MSB:MAMM:235838. *Marmosops ocellatus*: Santa Cruz: 15 km S of Santa Cruz, 17°53′S, 63°07′W, 2 August 1984, HWML118739 (2 specimens) from MSB:MAMM:58514. *Monodelphis domestica*: Chuquisaca: El Porvenir, 20°45′S, 63°13′W, 675 m, 7 July 1985, HWML60236 (1 specimen) from MSB:MAMM:55847. *Monodelphis sanctarosae*: Santa Cruz: Santa Rosa de la Roca, 15°30′00″S, 61°16′12″W, 250 m, 6 June 1990, HWML118802 (2 specimens) from MSB:MAMM:237023. *Philander opossum*: Santa Cruz: San Miguel Rincón, 17°22′59″S, 63°31′59″W, 300 m, 14 August 1984, HWML118740 (1 specimen) from MSB:MAMM:210528; 6 km by road W Ascención, 15°25′47″S, 63°53′59″W, 240 m, 13 August 1985, HWML118741 (6 specimens) from MSB:MAMM:211436.

*Additional specimens examined*: CHIOC12 (holotype) from *Didelphis aurita*, Rio de Janeiro Brazil.

CHIOC18356, CHIOC19115, from *Didelphis azarae*, Puerto Ibapobó, Paraguay. CHIOC4446 from *Tolypeutes tricninctus* (L.) Tanque, Brazil. CHIOC31879 from *Nectomys squamipes* Formosa, Goiás, Brazil. USNPC8550 from *Didelphis virginiana*, Houston, Texas, U.S. A.CMNA408 from *Didelphis marsupialis*, Saint Vincent, Trinidad, Trinidad and Tobago. CNHE2110 from *Didelphis marsupialis*, Motozintla, Mexico.

#### Superfamily Cosmocercoidea Railliet, 1916 Family Kathlaniidae Lane, 1914 *Cruzia* Travassos, 1917 22. *Cruzia tentaculata* (Rudolphi, 1819) Travassos, 1917

*Synonyms: Ascaris tentaculata* Rudolphi, 1819; *Oxysoma tentaculata* Schneider, 1866

*Site of infection*: Large intestine and caecum

*Type host and locality: Didelphis marsupialis*, Brazil

*Other reported hosts*: *Didelphis albiventris*, *Didelphis aurita*, *Didelphis pernigra, Didelphis virginiana*, *Metachirus nudicaudatus*, *Metachirus myosurus*, *Philander opossum*, *Philander quica* (Temminck).

*Locality Records*: Brazil: Bahía: Igrapiúna; Minas Gerais: Belo Horizonte, Conceição dos Ouros; Paraibá: Santa Rita; Paraná: Curitiba, Ponta Grossa; São Paulo: São Paulo; Rio Grande do Sul: Porto Alegre; Rio de Janeiro: Glicério, Barra de Marica, Casimiro de Abreu, Serra dos Orgãos, Sumidouro, Petrópolis; Santa Catarina: Santa Catarina Island; Sergipe: Capela, São Cristovão. Colombia: Valle del Cauca: Meléndez. Mexico: Chiapas; Colima; Distrito Federal; Estado de México; Hidalgo: Tasquillo; Guerrero; Jalisco; Morelos; Oaxaca; Veracruz; Yucatán. Peru: Ancash: Marca, Huanchoc; Loreto: Iquitos; Piura: valle del Huancabamba; San Martín: Bella Vista; Cajamarca: Cajamarca. United States: Louisiana, North Carolina, Pennsylvania, Tennessee, Texas, Wisconsin (Alden, 1995; Silva and Costa, 1999; Monet-Mendoza *et al*., 2005; Chero *et al*., 2017; Polo-Gonzales *et al*., 2019; Cirino *et al*., 2020).

*Records in Bolivia*: *Didelphis albiventris*: Tarija: Tapecua, 21°06′S, 63°55′W, 1500 m, 14 July 1991, HWML 118741 (39 specimens) from MSB:MAMM:239823; La Paz: Saynami Rio Zongo, 16°07′39″S, 68°05′59″W, 4 June 1993, HWML118742 (153 specimens) from MSB:MAMM:236299. *Didelphis pernigra*: La Paz: Yanacachi, Valle Aceromarka 16°19′35″S, 67°53′21″W, 3085 (Mollericona and Nallar, 2014). *Philander opossum*: Santa Cruz: 6 km by road W Ascención, 15°25′47″S, 63°53′59″W, 240 m, 13 August 1985, HWML118743 (99 specimens) from MSB:MAMM:211436.

*Remarks*: The species has an almost continental distribution and it is known to occur in armadillos and opossums (Ruiz, 1947; Fujita *et al*., 1995; Adnet *et al*., 2009; Souza *et al*., 2022). The material of this species across its putative range needs to be reviewed.

#### Suborder Spirurina Railliet and Henry, 1915 Superfamily Rictularioidea Hall, 1913 Family Rictulariidae Hall, 1913 *Pterygodermatites* Wedl, 1861 23. *Pterygodermatites* (*Paucipectines*) *elegans* (Travassos, 1928) Quentin, 1969

*Synonyms: Rictularia elegans* Travassos, 1928

*Site of infection*: Small intestine

*Type host and locality: Eumops perotis* (Schinz): Engenheiro Gomide, São Paulo, Brazil.

*Other reported hosts*: *Marmosa cinerea, Marmosa demerarae*.

*Locality Records:* Brazil: Cafezal, Belém. French Guiana: Macouria, Montagne du Tigre, Pic Matecho, Saül (Byles *et al*., 2013).

*Records in Bolivia*: *Marmosa* sp.: Santa Cruz: Estancia Cachuela Esperanza, 16°46′59.99″S, 63°13′59.99″W, 300 m, 24 August 1984, HWML60078 (15 specimens) from MSB:MAMM:211050; La Paz: Chijchijpa: 16°09′S, 67°45′W, 1114 m, 8 July 1992, HWML118744 (1 specimen) from host MSB:MAMM:235553. *Marmosops bishopi*: La Paz: La Reserva, 15°44′S, 67°31′W, 850 m, 27 July 1992, HWML118745 (1 specimens) from MSB:MAMM:235887.

*Additional specimens examined*: HWML67202 from *Marmosa demerarae*, Montagne du Tigre, Cayenne, French Guiana.

#### 24. *Pterygodermatites* (*Paucipectines*) *jaegerskioldi* (Lent and Freitas, 1935) Quentin, 1969

*Site of infection*: Small intestine

*Type host and locality: Caluromys philander* (L.), Rio de Janeiro, Tijuca, Brazil.

*Other reported hosts*: *Gracilinanus agilis, Gracilinanus microtarsus* (Wagner).

*Locality records:* Brazil: Mato Grosso do Sul, Nhecolândia; Rio de Janeiro: Parque Nacional da Serra dos Orgãos (Lent and Freitas, 1935; Torres *et al*., 2007, 2009).

*Records in Bolivia*: *Monodelphis domestica*: Santa Cruz: 27 km S of Santa Cruz, 3 km E and 1 km S Brecha Tres, 18°01′59″S, 63°10′01″W, 20 June 1992, HWML118746 (1 specimen) from MSB:MAMM:67022; 1 km S and 3 km W of Estancia Isibolos, 19°31′S, 63°36′, 930 m, 5 July 1991, HWML118747 (5 specimens) from MSB:MAMM:239734.

#### Superfamily Spiruroidea Oerley, 1885 Family Spiruridae Oerley, 1885 *Spirura* Blanchard, 1849 25. *Spirura guianensis* (Ortlepp, 1924) Chitwood, 1938

*Site of infection*: Stomach

*Type host and locality:* Monki monki (Scientific name not disclosed), Suriname.

*Other reported hosts*: *Didelphis marsupialis*, *Gracilinanus agilis*, *Marmosa cinerea*, *Marmosa demerarae*, *Marmosa murina*, *Metachirops opossum*, *Philander opossum, Saguinus geoffroyi* (Pucheran), *Saguinus nigricollis* (Spix), and *Tamarinus nigricollis* (Spix)

*Locality Records:* French Guiana: Montagne du Tigre. Brazil: Rio de Janeiro, Itaguaí; Mato Grosso do Sul, Nhecolândia. Panama: Panama Canal (Torres *et al*., 2009; Byles *et al*., 2013).

*Records in Bolivia*: *Chironectes minimus*: La Paz: La Reserva, 15°44′S, 67°31′W, 850 m, 22 July 1992, HWML118748 (34 specimens) from MSB:MAMM:68330. *Monodelphis domestica*: Chuqisaca: El Porvenir, 20°27′W, 63°07′48″S, 675 m, 15 July 1985, HWML 118756 (1 specimen) from host MSB:MAMM:211199

#### Family Gongylonematidae Hall, 1916 *Gongylonemoides* Lent and Freitas, 1937 26. *Gongylonemoides marsupialis* (Vaz and Pereira, 1934) Freitas and Lent, 1937

*Synonym: Gongylonema marsupialis* Vaz and Pereira, 1934

*Site of infection*: Esophagus

*Type host and locality: Didelphis aurita*, São Paulo, Brazil

*Other reported hosts*: *Didelphis aurita, Metachirops opossum*.

*Locality Records*: Brazil: Rio de Janeiro; São Paulo: undetermined. Peru: San Martín: Llamas (Gomes *et al*., 2003; Chero *et al*., 2017).

*Records in Bolivia*: *Marmosa* sp.: La Paz: Chijchijpa, 16°09′S, 67°45′W, 1114 m, 8 July 1992, HWML118749 (55 specimens) from host MSB:MAMM:235553; *Thylamys venustus*: Tarija: 3 km SE Cuyambuyo, 22°16′S, 64°33′W, 900 m, 4 August 1991, HWML118750 (3 specimens) from MSB:MAMM:140296.

#### Superfamily Physalopteroidea Railliet, 1893 Family Physalopteridae Railliet, 1893 *Turgida* Travassos, 1920 27. *Turgida turgida* (Rudolphi, 1819) Travassos, 1919

*Synonyms*: *Physaloptera turgida* Rudolphi, 1819; *Spiroptera turgida* Dujardin, 1845; *Physaloptera didelphidis* Leidy, 1851.

*Site of infection*: Stomach

*Type host and locality:* Brazil

*Other reported hosts*: *Caluromys derbianus*, *Didelphis aurita*, *Didelphis albiventris*, *Didelphis marsupialis* and *Didelphis virginiana*, *Metachirus nudicaudatus*, and *Philander opossum.*

*Locality Records*: Argentina: Santiago del Estero. Brazil: Goías: Nerópolis; Río de Janeiro: Casimiro de Abreu, Angra dos Reis, Sumidouro; Santa Catarina: Santa Catarina Island. Mexico: Chiapas: Motozintla, Tonalá; Colima: Colima, Comala, Dos Amates, La Esperanza, Madrid; Distrito Federal; Estado de México: Tequesquinahuac; Guerrero: Coyuquilla, Taxco El Viejo; Hidalgo: Tasquillo; Jalisco: Chamela; Michoacán, El Hortigal; Morelos; Oaxaca: Temazcal; Veracruz, Los Tuxtlas. Panama: Panama Canal. Peru: Loreto: Iquitos; Piura: San Felipe de Vichayal; San Martín: Bella Vista. United States: California, Connecticut, Colorado, Florida, Georgia, Illinois, Kansas, Louisiana, Oklahoma, New York, North Carolina, Pennsylvania, Tennessee, Texas, Virginia, Wisconsin. Trinidad and Tobago. Venezuela: Maracaibo (Alden, 1995; Monet-Mendoza *et al*., 2005; Chero *et al*., 2017; Polo-Gonzales *et al*., 2019).

*Records in Bolivia*: *Didelphis albiventris*: Tarija: Tapecua, 21°06′S, 63°55′W, 1500 m, 14 July 1991, HWML118751 (69 specimens) from MSB:MAMM:239823; *Philander opossum*: Pando: Bella Vista, 11°13′48″W, 67°07′12″W, 170 m, 26 July 1986, HWML118752 (2 specimens) from MSB:MAMM:211891.

#### Suborder Oxyurinae Railliet, 1895 Superfamily Oxyuroidea Cobbold, 1864 Family Oxyuridae Cobbold, 1864 *Didelphoxyuris* Gardner and Hugot, 1995 28. *Didelphoxyuris thylamisis* Gardner and Hugot, 1995

*Site of infection*: Large intestine and caecum

*Type host and locality: Thylamys venustus*: Santa Cruz, 5 km NE Quiñe, 18°03′S, 64°19′W, 1900 m, 27 May 1991.

*Other reported hosts*: Other than symbiotype, none available

*Locality records*: Other than type locality, none available

*Records in Bolivia*: *Marmosa* sp.: Santa Cruz: 53 km E Boyuibe, 20°27′S, 62°50′W, 600 m, 6 July 1991, HWML118755 (12 specimens) from MSB:MAMM:239772. *Thylamys venustus*: Tarija: 3 km SE Cuyambuyo, 22°16′S, 64°33′W, 900 m, 4 August 1991, HWML117853 from MSB:MAMM:140296, HWML117854 (256 specimens) from MSB:MAMM:140297; Tarija: Tapecua, 21°26′S, 63°55′W, 1500 m, 12 July 1991, HWML61315 (17 specimens) from AMNH275439; Santa Cruz: 5 km NE Quiñe, 18°03′S, 64°19′W, 1900 m, 27 May 1991, HWML61086 NK22813 MSB:MAMM:87107 (360 specimens). *Thylamys pusillus*: Santa Cruz: 53 km E Boyuibe, 20°27′S, 62°50′W, 600 m, 6 July 1991, HWML61267 (28 specimens) from MSB:MAMM:87105

*Additional specimens examined*: HWML39072 holotype

*Remarks*: The symbiotype was originally identified as *Thylamys elegans* Waterhouse. However, a systematic review of the genus revealed that this species is restricted to the western slope of the Andean Cordillera; furthermore, fat-tailed opossusms infected with pinworms used in the species description belong to *Thylamys venustus* (Giarla *et al*., 2010).

#### *Monodelphoxyuris* Guerrero and Hugot, 2003 29. *Monodelphoxyuris dollmeiri* Guerrero and Hugot, 2003

*Site of infection*: Large intestine and caecum

*Type host and locality*: *Monodelphis emiliae* (Thomas): San Martín, Rio Camisea, Cusco, Peru, 11°47′10″S, 72°42′5″W, 474 m; May 08, 1997. Accession number CHIAUMSM1175. Symbiotype 14149 Mammal Collection of the Museo de Historia Natural, Universidad Nacional Mayor de San Marcos, Lima.

*Other reported hosts*: None available

*Locality records*: Other than type locality, none available

*Records in Bolivia*: *Monodelphis domestica*: Chuqisaca: El Porvenir, 20°27′W, 63°07′48″S, 675 m, 15 July 1985, HWML 118756 (158 specimens) from host MSB:MAMM:211199; HWML 60229 (88 specimens) from host AMNH M 261233. *Thylamys venustus*: Chuqisaca: El Porvenir, 20°27′W, 63°07′48″S, 675 m, 13 July 1985, HWML 60130 (2 specimens) from host MSB:MAMM:211181

#### *Neohilgertia* Navone, Suriano and Pujol, 1990 30. *Neohilgertia venusti* Navone, Suriano and Pujol, 1990

*Site of infection*: Large intestine and caecum

*Type host and locality: Thylamys venustus*, Tucuman, Burruyacu. Bernardino Rivadavia Helminthological Collection 362.

*Other reported hosts*: None available

*Locality records*: Other than type locality, none available

*Records in Bolivia*: *Thylamys* sp.: Tarija: 3 km S of Cuyambuyo, 22°16′S, 64°33′W, 900 m, 4 and 5 August 1991, HWML61553 (36 specimens) from MSB:MAMM:240043; HWML118792 (42 specimens) from MSB:MAMM:240056

#### Class Dorylaimea Hodda, 2007 Order Trichocephalida Spasski, 1954 Family Trichuridae Ransom, 1911 *Trichuris* Roederer, 1761 31. *Trichuris reesali* Wolfgang, 1951

*Site of infection*: Large intestine and caecum

*Type host and locality: Didelphis marsupialis*, Trinidad

*Other reported hosts*: *Didelphis marsupialis*, *Marmosa demerarae*, *Marmosa murina, Philander opossum* (Wolfgang, 1951; Byles *et al*., 2013).

*Locality Records*: French Guiana: Camp du Tigre, Saül, Macouria.

*Records in Bolivia*: *Marmosa* sp.: Santa Cruz: Estancia Cachuela Esperanza, 16°46′59.99″S, 63°13′59.99″W, 300 m, 24 August 1984, HWML118807 (2 specimens) from MSB:MAMM:211050; *Marmosops noctivagus*: La Paz: La Reserva, 15°44′S, 67°31′W, 850 m, 24 July 1992, HWML118757 (7 specimens) from MSB:MAMM:235815.

### Phylum Acanthocephala Rudolphi, 1808

#### Class Archiacanthocephala Meyer, 1931 Order Moniliformida Schmidt, 1972 Family Oligoacanthorhynchidae Southwell and McPhie, 1925 *Oligacanthorhynchus* Travassos, 1915 32. *Oligacanthorhynchus microcephalus* (Rudolphi, 1819) Schmidt, 1972

*Synonyms: Echinorhynchus microcephalus* Rudolphi, 1819; *Echinorhynchus tortuosa* Leidy, 1850; *Hamanniella microcephalus* (Rudolphi, 1819) Travassos, 1915; *Hamanniella tortuosa* (Leidy, 1850) Van Cleave, 1924; *Hamanniella tumida* (Van Cleave, 1947) Van Cleave, 1953; *Oligacanthorhynchus tortuosa* (Leidy, 1850) Schmidt, 1972; *Oligacanthorhynchus tumida* (Van Cleave, 1947) Schmidt, 1972; *Travassosia tumida* Van Cleave, 1947

*Site of infection*: Small intestine

*Type host and locality: Caluromys philander*, Brazil

*Other reported hosts*: *Didelphis albiventris*, *Didelphis marsupialis*, *Didelphis virginiana*, *Marmosa demerarae*, *Marmosa murina, Metachirus myosurus*, *Philander opossum. Dasypus novemcinctus* L., *Euphractus sexcinctus* (L.).

*Locality Records*: Brazil: Bahía: Igrapiúna; Rio de Janeiro: Rio de Janeiro; São Paulo: São Paulo. Colombia: Chocó. Mexico: Campeche, Colima, Michoacán, Guanajuato, Morelos, Oaxaca, Tabasco, Veracruz, Yucatán. French Guiana: Montagne du Tigre, Macouria, Pic Matecho. Paraguay: Chaco Boreal. Suriname. United States: Alabama, Arkansas, Florida, Georgia, Illinois, Louisiana, Mississippi, Oklahoma, South Carolina, Texas. Venezuela (Richardson *et al*., 2014; Acosta-Virgen *et al*., 2015; Cirino *et al*., 2020).

*Remarks: Oligacanthorhynchus microcephalus* was collected from *Metachirops opossum* and *Philander opossum* from Santa Cruz, Bolivia and used for a redescription of the species (Richardson *et al*., 2014).

## Discussion

Bolivia contains a large variety of biomes and it is rich in natural resources (Ergueta and Salazar, 1991; Auty, 1994; Anderson, 1997; Hancock *et al*., 2018). Among these resources, some minerals are used in high demand (Finer *et al*., 2008; Hancock *et al*., 2018), and vast areas of the country have been converted to agricultural use (Cuellar and Noss, 2014). The exploitation of these natural resources wipes out natural habitats and causes extinction of native biodiversity (Finer *et al*., 2008; Cuellar and Noss, 2014; Gardner *et al*., 2021). These abrupt modifications change the interactions among species occurring in the biome, which includes the dynamics that regulate the interactions between parasites and hosts (Gardner and Campbell, 1992*b*). The parasite checklist herein presented is a historical document that summarizes the marsupial parasite association present in Bolivia by the end of the Twentieth Century.

Of the 35 species of marsupials recorded for Bolivia we document the helminthological record for 17. Most of the specimens were collected in localities across the Chaco, and Yungas, with few individuals collected from the Amazon basin. The majority of the specimens representing both parasites and hosts were preserved and archived in relevant repositories for biodiversity, which include the Harold W. Manter Laboratory of Parasitology (Lincoln, Nebraska), the Museum of Southwestern Biology (Albuquerque, New Mexico), and the American Museum of Natural History (New York, New York). The records resulted from the synergistic effort of mammalogists and parasitologists participating in the Bolivian Faunistic Inventories completed by the end of the previous century (Anderson, 1997). The present checklist includes the helminths collected from didelphiomorphs and it expands on other published checklists that document the diversity of parasites in mammals present in Bolivia (Dick *et al*., 2007; Pucu *et al*., 2014; Sanchez *et al*., 2018).

A greater effort is necessary to complete the inventory of the parasites of marsupials present in Bolivia, since 17 species of didelphiomorphs, plus *Lestoros inca* (Thomas), representative of Coenolestidae, have not been surveyed for helminths. Furthermore, the assessment of the helminth fauna associated with each species may be hindered by the relatively large sample size necessary to survey the parasite species richness in marsupials (Jiménez *et al*., 2011; Byles *et al*., 2013).

Nematodes represent the most diverse group in this checklist; it includes 22 species of which 9 are included in Viannaiidae and only occur in didelphiomorph marsupials. The second largest group includes cyclophyllidean tapeworms (6 species) followed by rhopaliid trematodes (3 species) and 1 species of acanthocephalan. From the total of parasite species, 16 are monoxenous, and include nematodes of the Viannaiidae, Oxyuridae, Trichuridae, Molineidae and Ancylostomatidae. The other 16 species are heteroxenous, and they depend on molluscs or insects to infect their definitive hosts. Heteroxenous species may be used as indicators of the local biodiversity by revealing the taxonomic and trophic levels that still function in any given locality (Gardner and Campbell, 1992*b*). A modest fraction of the organisms used in this checklist have served as the foundation for systematic reviews and species descriptions for trematodes, cestodes and nematodes (Gardner and Campbell, 1992*a*; Haverkost and Gardner, 2008; Jiménez *et al*., 2008; Gardner *et al*., 2013), underscoring the relevance of species descriptions as the sole records documenting biodiversity.

As a consequence, the present checklist incorporates additional localities that better represent the distribution of parasites and their host spectrum. The results listed in this checklist should act as a starting point to build upon the diversity of mutualists, micropredators or parasites associated with marsupials. As stated elsewhere (Gardner and Campbell, 1992*b*; Wood *et al*., 2023), the changes in the quality of the habitat and the diversity of organisms will determine the likelihood of heteroxenous species to complete their life cycle.

The identification of helminths herein presented documents their distribution in nearly 50% of the marsupial biodiversity of Bolivia. None of the species included in this checklist are known to have a zoonotic potential. Nevertheless this list should complement the efforts to screen these organisms for microparasites, which may be of zoonotic relevance and have been documented across Bolivia (Messenger *et al*., 2015). This is important because several species of marsupials are synanthropic and thrive in human altered environments (Bezerra-Santos *et al*., 2021; Voss, 2022). This innate ability makes it important to continue the surveillance of parasites in these mammals, since they can expose human populations to unanticipated outbreaks. The surveillance is an important component of proposed novel protocols to prevent outbreaks resulting from anthropogenic alterations (Hoberg *et al*., 2022; Gardner *et al*., 2023).

## Data Availability

The helminthological record for most of the marsupials examined is available at http://opensiuc.lib.siu.edu/zool_data/23/.
